# Multinodular and Vacuolating Neuronal Tumor: Incidental Diagnosis of a Rare Brain Lesion

**DOI:** 10.7759/cureus.20674

**Published:** 2021-12-25

**Authors:** Sophia Arbuiso, Katie Roster, Amanpreet Gill, Omar Tarawneh, Kyril L Cole, Syed Faraz Kazim, John Vellek, Meic H Schmidt, Christian A Bowers

**Affiliations:** 1 School of Medicine, New York Medical College, Valhalla, USA; 2 College of Osteopathic Medicine, Burrell College of Osteopathic Medicine, Las Cruces, USA; 3 Department of Neurosurgery, Univesity of Utah, Salt Lake City, USA; 4 Department of Neurosurgery, University of New Mexico School of Medicine, Albuquerque, USA

**Keywords:** incidental radiological finding, asymptomatic., benign brain tumor, dysembryoplastic neuroepithelial tumor (dnet), multinodular and vacuolating neuronal tumor (mvnt)

## Abstract

Multinodular and vacuolating neuronal tumor (MVNT) is a rare benign brain lesion, commonly found in middle-aged adults. The patients experience a range of symptoms from being asymptomatic to epileptic seizures, with headache being the most common symptom. Here we report a case of an incidental diagnosis of MVNT in a young female. A 25-year-old female with a past medical history of occasional headaches without seizures or any focal neurological deficit presented after a motor vehicle rollover. The MRI brain revealed an incidental finding of a subcortical lesion in the right parietal lobe with T2-FLAIR (fluid-attenuated inversion recovery) hyperintensity between the cystic portions, indicative of a possible MVNT, with a less probable chance of dysembryoplastic neuroepithelial tumor based on the subcortical location of the lesion. No neurosurgical intervention was recommended. With one-year follow-up, no changes were noted on neuroimaging, and the patient remained stable without any neurological symptoms. The MVNT is a rare brain lesion that presents with benign features. In patients with epileptic symptoms, surgical resection of the lesion can be curative. However, in asymptomatic patients, careful monitoring may be sufficient, as described in this case.

## Introduction

In 2013, the first documented case of multinodular and vacuolating neuronal tumors (MVNTs) was reported [1]. In 2016, MVNT was classified by the World Health Organization Classification of Tumors of the Central Nervous System [2]. Most of the MVNT cases reported yet have been in middle-aged patients with neurological symptoms including headaches and seizures [3-6]. Surgical intervention is often indicated in MVNTs related to epileptic symptoms [3-6]. However, asymptomatic cases of MVNT have rarely been reported, suggesting MVNT may go undiagnosed, and therefore surgical intervention is infrequently necessary [5]. Here we present the case of a 25-year-old female patient with incidental finding of an MVNT with no neurologic deficits.

## Case presentation

**Figure 1:**
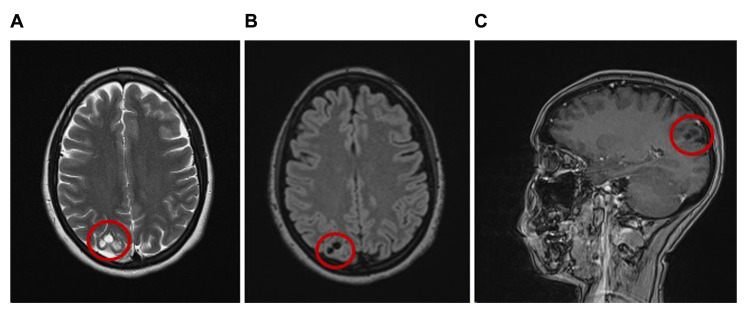
MRI brain with and without contrast of MVNT patient presented in this case report MRI brain with and without contrast at initial presentation revealed the lesion identified in the subcortical white matter in the right parietal lobe with areas of more solid T2-FLAIR hyperintensity between the cystic processes, consistent with MVNT. The axial T2 (A), axial T2-FLAIR (B), and sagittal T1 post-contrast (C) images are shown. MVNT, multinodular and vacuolating neuronal tumor; FLAIR, fluid-attenuated inversion recovery.

A 25-year-old female with a past medical history of hepatitis C, heroin abuse, and depression was admitted after a motor vehicle accident that resulted in multiple fractures. An MRI brain with and without contrast done at initial presentation revealed an incidental finding of a subcortical lesion in the right parietal lobe with T2-FLAIR (fluid-attenuated inversion recovery) hyperintensity between the cystic portions indicating a possible MVNT, with a less probable chance of dysembryoplastic neuroepithelial tumor based on lesion location (Figure 1A-1C).

At the time of initial presentation, the patient did not report any visual disturbances, seizures, nausea, vomiting, or other neurological deficits. She had a past medical history of occasional headaches responding to over-the-counter pain medications. She had no past medical history of any neurological condition. The patient was identified to have abnormal brain lesion on neuroimaging as a child too; however, this could not be verified due to inability to obtain her childhood scans. Neuroimaging at her three-month, six-month, and one-year follow-ups showed no change in the appearance of the brain lesion; also, the patient continued to have no neurological symptoms except for occasional headaches. Surgical intervention was therefore not recommended.

## Discussion

First reported in 2013, MVNTs are sporadic benign brain tumors [1,3,6]. MVNTs have been associated with ganglion cell tumors found in the cortical white matter [3]. Very few cases of MVNT have been reported so far. Fukushima and colleagues described a surgically resected MVNT containing proliferated neuronal cells, some of which were ganglion-like cells with the presence of HuC/HuD [4]. Another report, including 16 cases of MVNT, found MVNTs to be twice as common in females, with an average age of 42 years at diagnosis; of the 16 cases, 10 had tumors located in the left hemisphere, with 87.5% located in the frontal and parietal lobes [3]. Conversely, Huse and colleagues found seven out of 10 MVNTs in their series to be in the temporal lobe [1].

Surgical resection of MVNTs is discouraged for asymptomatic cases, only to be recommended when seizures are definitively linked to the MVNT [1,3-6]. A definitive diagnosis can only be made on biopsy; however, there are significant risks with the invasive procedure, considering most of these MVNT lesions’ locations. Instead, reliable MRI brain findings have been documented that can instead be used to diagnose MVNT lesions, such as hyperintensity seen in FLAIR and T2-weighted images [1,3-6]. Importantly, in the limited cases' data available yet, MVNTs typically do not change on follow-up imaging.

Here we present a case that is particularly rare due to several factors. With our patient being 25 years old, she was much younger than the average patient diagnosed with MVNT [1,3-6]. Moreover, there were hints that this tumor may in fact be present in her brain from a young age due to the history of an abnormal lesion observed on earlier neuroimaging as a child; however, this could not be confirmed. Our case is also particularly unique given the asymptomatic presentation of our patient. Previously, MVNT with no neurological symptoms was reported in a 60-year-old man [6]. Additionally, MVNT was reported in a 33-year-old female diagnosed with epilepsy following an episode of limbic encephalitis two years prior [5]. In that case, the tumor was identified because of the patient’s seizures, localized to the temporal lobe; however, it was concluded that the seizures were unrelated to the MVNT [5]. Consistent with the previous reports, our patient’s MVNT showed no change on imaging at three-month, six-month, and one-year follow-up. The present report seeks to contribute to the limited knowledge available on MVNTs to the medical community.

## Conclusions

MVNT is a rare, benign brain lesion that tends to remain unchanged over time. MVNTs have reliable radiologic findings; however, these tumors have varying presentation related to the location in the brain. Here we described a case of an asymptomatic MVNT in a 25-year-old female, younger than the widely reported age of MVNT diagnosis. Over the available follow-up period, the patient’s tumor remained unchanged since the initial incidental finding. The present report adds to the limited number of MVNT cases with radiological features reported in neurosurgical literature.
